# The Roles of NEDD4 Subfamily of HECT E3 Ubiquitin Ligases in Neurodevelopment and Neurodegeneration

**DOI:** 10.3390/ijms23073882

**Published:** 2022-03-31

**Authors:** Shanez Haouari, Patrick Vourc’h, Médéric Jeanne, Sylviane Marouillat, Charlotte Veyrat-Durebex, Débora Lanznaster, Frédéric Laumonnier, Philippe Corcia, Hélène Blasco, Christian R. Andres

**Affiliations:** 1 UMR 1253, iBrain, Université de Tours, Inserm, 37044 Tours, France; shanez.haouari@etu.univ-tours.fr (S.H.); mederic.jeanne@univ-tours.fr (M.J.); sylviane.marouillat@univ-tours.fr (S.M.); c.veyratdurebex@chu-tours.fr (C.V.-D.); debora.lanznaster@univ-tours.fr (D.L.); frederic.laumonnier@univ-tours.fr (F.L.); philippe.corcia@univ-tours.fr (P.C.); helene.blasco@univ-tours.fr (H.B.); andres@med.univ-tours.fr (C.R.A.); 2 CHRU de Tours, Service de Biochimie et Biologie Moléculaire, 37044 Tours, France; 3 CHRU de Tours, Service de Génétique, 37044 Tours, France; 4 CHRU de Tours, Service de Neurologie, 37044 Tours, France

**Keywords:** ubiquitin, ligases, development, neurodegenerative, intellectual disability, ALS

## Abstract

The ubiquitin pathway regulates the function of many proteins and controls cellular protein homeostasis. In recent years, it has attracted great interest in neurodevelopmental and neurodegenerative diseases. Here, we have presented the first review on the roles of the 9 proteins of the HECT E3 ligase NEDD4 subfamily in the development and function of neurons in the central nervous system (CNS). We discussed their regulation and their direct or indirect involvement in neurodevelopmental diseases, such as intellectual disability, and neurodegenerative diseases, such as Alzheimer’s disease, Parkinson’s disease or Amyotrophic Lateral Sclerosis. Further studies on the roles of these proteins, their regulation and their targets in neurons will certainly contribute to a better understanding of neuronal function and dysfunction, and will also provide interesting information for the development of therapeutics targeting them.

## 1. Introduction

The development and function of the central nervous system (CNS) are complex processes that require dynamic mechanisms including stages of proliferation, migration, differentiation, maturation and synaptic plasticity. These processes are finely regulated thanks to the involvement of numerous proteins with varied cellular localizations and roles [1] Control of the concentrations of these proteins, named cellular protein homeostasis (proteostasis), is achieved at the level of their synthesis and their degradation. Control of the function of these proteins is also crucial for correct development and functioning of the CNS. An important part of this control is realized by dynamic post-translational modifications. The ubiquitin (Ub)/Ub-like systems consist of one of them [2]. Genes encoding members of these two systems are directly implicated in neurodevelopmental disorders including syndromic and non-syndromic intellectual disabilities (*RNF12*, *CUL4B*), and neurodegenerative diseases, such as Parkinson’s disease (*PARK2*) or Amyotrophic Lateral Sclerosis (*CCNF*) [3,4,5].

The Ub/Ub-like systems consist of intracellular pathways comprising 3 classes of enzymes, E1, E2 and E3 (RING or HECT families) encoded by over 700 genes in the human genome. These pathways work by adding one or more small ubiquitins (76 aa) or ubiquitin-like proteins (e.g., SUMO, Small Ubiquitin MOdifier) to target proteins. Depending on the post-translational modification, the tagged protein will be sent to the proteasome for degradation or will have its function regulated or modified [6]. The Ub/Ub-like systems have been extensively studied in recent years, but many questions still exist regarding their regulation and function, which proteins they target, and their roles in the pathophysiology of human diseases. The largest HECT E3 subfamily is the neuronal precursor cell-expressed developmentally downregulated 4 (NEDD4) subfamily. The objective of this review was to discuss the physiological role in the CNS of this NEDD4 subfamily, and its implication in neurodevelopmental and neurodegenerative diseases.

## 2. Overview of the Ubiquitin System

Ubiquitination is a key mechanism in protein degradation mediated by the proteasome. It consists of adding ubiquitins (Ub) onto target proteins. Ubiquitin is a 76 amino acids protein with 7 lysine residues used to link target protein or other Ub (for polyubiquitination) [7]. Ubiquitin protein can be bound to the target protein as a ubiquitin monomer (monoubiquitination) or as a ubiquitin polymer (polyubiquitination). A protein can also be multiubiquitinated, which consists of several monoubiquitinations of the target protein. Monoubiquitination and binding to a chain of ubiquitins linked by lysines-63, for example, are functional regulatory signals for the ubiquitinated protein. Binding to a chain of lysine-48-linked ubiquitins is a signal for degradation of the ubiquitinated protein by the proteasome. Ubiquitinated proteins can also be deubiquitinated by about a hundred deubiquitinating enzymes [8]. The diversity of actors of the ubiquitin system and the modifications it produces on proteins involve this system in many cellular processes, such as protein homeostasis, endocytosis, intracellular trafficking, cellular stress and autophagy [9,10,11,12,13].

**Figure 1:**
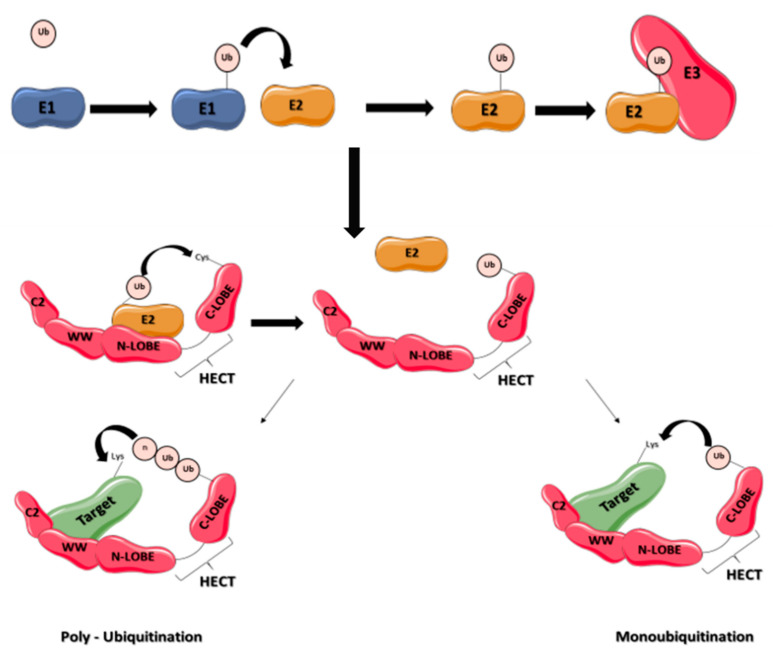
Schematic representation of ubiquitination process by E3 ligases containing HECT domain. Ubiquitin is activated by an enzyme E1 and then transferred to E2 enzymes, and to a HECT E3 enzyme. The E3 enzyme transfers ubiquitin to the target protein, leading to its monoubiquitination or polyubiquitination. The E3 ligase contains 3 domains, an N-terminal C2 domain, a WW rich domain and a C-terminal domain organized into two lobes (N-lobe and C-lobe).

Five percent of the genes in the human genome encode proteins of the ubiquitin system, which is impressive. Ubiquitination of proteins is achieved by a coordinated system of three classes of enzymes: ubiquitin-activating enzymes (E1), conjugating enzymes (E2) and ligases (E3). The single E1 enzyme activates ubiquitin for transfer [14]. The E2 enzymes, of which there are 38, directly transfer ubiquitin to the target proteins with the help of a third class of enzymes, the E3 ligases, which contain a Really Interesting New Gene (RING) domain [15]. This E3 ligases family is composed of over 600 members. The other families of E3 are the E3 with U-Box domain, the E3 with RING-between-RING (RBR) domain and the E3 with a Homologous to EA6P C-terminus (HECT) domain [8]. Some E2 enzymes transfer ubiquitin to HECT-E3 enzymes, which then transfer it to the target proteins (Figure 1). E3 ligases with HECT domain number are 28 in humans. Their size varies from 80 to 500 kDa. The common feature of these enzymes is the presence of a conserved C-Terminal, catalytic HECT domain of 350 amino acids residues [16]. HECT E3 ligases are classed into three subfamilies: NEDD4, HERC, and other HECT.

## 3. The NEDD4 E3 Ligases Subfamily

### 3.1. Structure and Diversity

**Figure 2:**
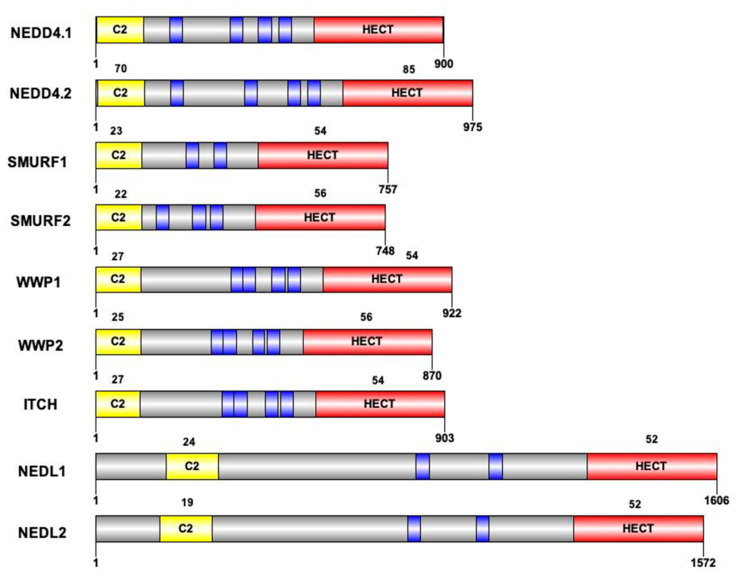
Structure of the 9 HECT E3 ligases of the NEDD4 subfamily. Protein size and domain conservation expressed as percentage of conservation compared to NEDD4.1 protein. Sequence of the nine members (UniprotKB) and identity percentage are obtained by alignment (MUSCLE) for the C2 and the HECT domains. Blue boxes represent the WW domains of each protein. Due to differences in the number of WW domains across proteins, identity percentage is not calculated for these domains.

The NEDD4 subfamily is found throughout eukaryotes. It includes the nine E3 ligases NEDD4-1, NEDD4-2, ITCH, WWP1, WWP2, SMURF1, SMURF2, NEDL1 and NEDL2 sharing a common structure characterized by the presence of 3 domains differently conserved during mammalian evolution: one N-terminal C2 domain, two to four tryptophan-tryptophan (WW) domains, and a HECT domain (Figure 1). The C2 domain has the ability to bind to phospholipids in a calcium-dependent manner. As a consequence, proteins containing C2 domain can mediate intracellular targeting to the plasma membrane, endosomes and multivesicular bodies [17]. It was first described in the protein kinase C (PKC) and is composed of 8 beta-strands that can coordinate two or three calcium ions. The WW domains, which contain on average 40 amino acids with two invariant tryptophan residues, are involved in protein/protein interactions. Interaction is done with proline-rich motifs (PY or PPxY) present on target proteins. Binding specificity of the WW domain comes from its β-stranded folding of the residues present in the loops that connect them. The C-terminal HECT domain is the E3 catalytic domain with the presence of a catalytic cysteine. It has a size of about 350 amino acids organized in two lobes, N-lobe and C-lobe. The N-lobe binds to E2 enzyme-ubiquitin complex, allowing transfer of the ubiquitin protein from the E2 to the C-lobe via a thioester intermediate with the catalytic cysteine. Once ubiquitin is transferred to the C-lobe, it can then be transferred to the target protein (Figure 1). These 3 domains were differently conserved during mammalian evolution (Figure 2).

### 3.2. Functions and Regulations

The 9 members of the NEDD4 subfamily bind an important number of target proteins and are implicated in a wide range of cellular processes. The proteins Neural precursor cell-expressed Developmentally Down-regulated protein 4 number 1 (NEDD4-1) and NEDD4 number 2 (NEDD4-2) were the first members to be discovered in this subfamily and are currently the most studied ones. NEDD4-1 is ubiquitously expressed and involved in many human cellular functions implicating multiple target proteins. Among these target proteins are PTEN, Akt, Beclin1 or FGFR1, which imply NEDD4-1 participation in cell proliferation, differentiation, migration and invasion, but also apoptosis, autophagy and DNA damage response. The subfamily funder member is thought to be implicated in various diseases, from cancers to neurodegenerative diseases [18]. NEDD4-2 is an E3 ligase for many ion channels, including sodium, chloride and potassium channels, such as the epithelial Na+ channel ENaC, which is implicated in sodium and fluid absorption in the lung, kidney and colon [19,20,21]. It regulates ENaC function by binding to its PY motifs, controlling the number of channels at the cell surface [22,23]. It also interacts with proteins implicated in the Wnt signaling pathway, TGF-β signaling pathway and autophagy [24,25,26]. The regulation of NEDD4-2 activity can be mediated by phosphorylation [27,28,29]. For example, its phosphorylation by RAC-alpha serine/threonine kinase protein 1 (AKT1) and serum and glucocorticoid-regulated kinase 1 (SGK1) leads to the recruitment of the adaptor protein 14.3.3 which interferes with ENaC [27]. The phosphorylation of NEDD4-2 also interferes with ORAI binding, which affects calcium signaling [30]. Interestingly, the C2 domain of NEDD4-1 and NEDD4-2 acts as an autoinhibitory domain of the E3 ligase activity. Calcium, by binding to the C2 domain, releases this autoinhibition.

The Itchy E3 Ubiquitin Protein Ligase Homolog protein (ITCH) controls a large spectrum of biological mechanism due to over 50 target proteins, including Jun proteins and the two members of p53 family, p63 and p73 [31,32,33]. ITCH plays a role in the regulation of TGF-β signaling pathway and acts on tumorigenesis [34,35]. It is implicated in other important pathways, such as Hedgehog, Hippo, Wnt and Notch signaling pathways [36,37,38,39]. It participate in endosomal and lysosomal functions, and DNA damage response [31,40,41]. Regulation of ICTH activity can be obtained by binding to proteins, such as N4BP1 which interacts with the WW2 domain of ITCH impeding interactions with its target protein (p73a, JUN, p63). Phosphorylation of ITCH by JNK1 activates the protein.

The WW-domain containing Protein 1 (WWP1) is a multifunctional protein with numerous targets, such as Smad2, Smad4, ErbB4/HER4, JunB and p53. Consequently, WWP1 has a role in transcription, protein trafficking, protein degradation, apoptosis and viral budding, among others. It has been implicated in cancers, such as colon and breast cancers, infectious diseases and neurological diseases [42]. It binds to its target proteins mainly by its WW domain. However, it binds to p53 independently of its WW domain. This interaction positively regulates and stabilizes p53 and thus activates apoptosis [43]. WWP2 is another E3 ligase of this subfamily that binds to targets involved in different signaling pathways, such as the PI3K/Akt or the TGF-β pathways. It also binds to and downregulates ENaC [44] and ubiquitinates the RNA polymerase II [45]. Due to its diverse functions, WWP2 has been implicated in cancers and in the modulation of the immune system [46].

The SMAD Ubiquitylation Regulatory Factor 1 and 2 (SMURF1 and SMURF2) were first discovered as E3 ligases capable of negative regulation of the TGF-β/BMP signaling pathways [47,48]. However, they are also implicated in other mechanisms. SMURF1 targets the noncanonical Wnt pathway and the MAPK pathway [49]. Accordingly, SMURF1 is involved in regulation of cell growth and morphogenesis, cell migration and polarity and in autophagy. Its catalytic activity can be enhanced by binding to casein kinase 2—interacting protein 1 (CKIP1) by its WW domain [50]. SMURF1 also regulates p53 activity, independently of its enzymatic activity, by binding to another E3 ligase, MDM2, whose ubiquitination activity will increase. MDM2 is a RING-E3 ligase that will target p53 for degradation by the proteasome [51]. SMURF2 is implicated in similar mechanisms. Its dual role in cancer is often discussed as it could act both as a tumor suppressor and activator. It is also involved in genomic stability by acting on chromatin and apoptosis [49].

The HECT, C2 and WW domain containing E3 ubiquitin protein ligase 1 (HECW1) and 2 (HECW2), also known as NEDD4-like ubiquitin protein ligase 1 (NEDL1) or 2 (NEDL2), are the most recently discovered members in the NEDD4 subfamily. Functional studies on these two proteins are just beginning. NEDL1 is involved in the Wnt signaling pathway by ubiquitination and degradation of Dishevelled-1 (Dvl1) [5,52,53]. Recent findings support that NEDL1 is also implicated in the TGF-β signaling pathway by ubiquitination of Smad4 [54]. These two proteins, HECW1 and HECW2, seems to interfere in various physiological mechanism such as enteric nervous system and kidney development [55,56].

As described above, the NEDD4 E3 ligases subfamily can be regulated in various ways. These enzymes can bind to various proteins via interactions with their three domains, leading to positive or negative regulation. An example of positive regulation is the action of adaptor proteins, such as Smads, that facilitate binding of substrates of TGF-beta pathway on the two SMURF proteins, NEDD4-2, WWP1 and ITCH ligases. [47,57,58,59,60,61]. NEDD4 family interacting protein 1 and 2 (NDFIP1 and NDFIP2) also facilitates the action of ITCH and NEDD4-1 [62,63]. WW linkers peptides, small sequences between two WW regions, interact with the HECT catalytic domain in these E3 ligases, leading to self-regulation of the NEDD4 subfamily proteins. These interactions prevent the activity of the catalytic domain and sometimes drive to autoubiquitination [64]. NEDD4 subfamily members can also be regulated by post-translational modifications. We have already mentioned phosphorylation for NEDD4-2, for example. Studies also observed SUMOylation of SMURF2, and neddylation of SMURF ligases, ITCH, NEDL1 and NEDL2 [65].

## 4. NEDD4 E3 Ligases in Neurodevelopment

Many studies support that E3 ubiquitin ligases play crucial roles in CNS development, from proliferation of stem cells and progenitors to neuronal differentiation, maturation and functioning [2,66]. NEDD4 subfamily members appear to be actively involved in these various stages of CNS development.

The first stage of CNS development is the proliferation of undifferentiated brain cells. Several cell signaling pathways are strongly involved in this stage, such as the bone morphogenetic protein BMP, TGF-β and Wnt signaling pathways, all of which are regulated in part by the HECT E3 SMURF1 and SMURF2, as described above [48,57,67]. NEDD4-1 is known to promote cell proliferation [68], as is WWP2, whose silencing significantly reduces the cell proliferation rate in vitro [69]. NEDD4-1 binds, by its third WW domain, the non-canonical sequence (non-PY motif) of FGFR1, resulting in its ubiquitination [70]. The FGF/FGFR1 (fibroblast growth factor/receptor 1) signaling pathway consists of another important pathway for CNS development. It is necessary for hippocampal growth in the CNS, for example, because it promotes the proliferation of hippocampal progenitors and stem cells during development in mice [71]. Maintenance of the neural stem cell pool and self-renewal also requires the Hedgehog signaling pathway. The Hedgehog transcription factor Gli1 is targeted by the protein Numb for ITCH-dependent ubiquitination, which suppresses the Hedgehog signal [72]. It is interesting to note that truncating mutations in ITCH have been identified in children with multisystem autoimmune diseases, dysmorphic features, relative macrocephaly and neurodevelopmental abnormalities including developmental delay and cognitive impairment [73].

The second stage of CNS development consists of migration of the cells in the brain and spinal cord, and their differentiation into specific types of neurons and glial cells. WWP1 and WWP2 knockout results in defects in axon–dendrite polarity in pyramidal neurons and in aberrant laminar cortical distribution showing that these NEDD4-like E3 ligases are essential for proper polarization of developing neurons [74]. SMURF1, by regulation of the Rho GTPase, promotes neurite outgrowth [75]. Moreover, its phosphorylation on Threonine 306 by the protein kinase A promotes axon formation. Preventing this phosphorylation results in altered polarization in cortical neurons in vivo [76]. NEDD4-1/small GTPase Rap2A signaling pathway regulates neurite growth and arborization in neurons [77]. NEDD4-2 also promotes axonal growth [78]. Genetic variants in *NEDD4-2* have been observed in patients with periventricular nodular heterotopia, polymicrogyria, macrocephaly, cleft palate, and syndactyly [79], suggesting a role for NEDD4-2 in neuronal migration. HECW2 consists of another important NEDD4 HECT E3 ligase in neurodevelopment. It stabilizes p73, [80] a crucial factor for neurogenesis and neurodevelopment. Mice lacking p73 expression show severe neurodevelopmental abnormalities with hippocampal dysgenesis [81]. Recently, de novo mutations in the *HECW2* gene have been identified in patients with neurodevelopmental diseases, including epilepsy, intellectual deficiency and macrocephaly [82,83,84,85].

The third stage of CNS development involves the formation of innumerable connections among neurons, both within and across regions. Phosphatase and tensin homolog (PTEN) is a known target of NEDD4-1 for ubiquitination, followed by degradation. The interaction between PTEN and NEDD4-1 seems to be implicated in the building of synaptic connections. NEDD4-1 is expressed in Xenopus retinal ganglion cells, where dysfunction of the E3 ligase leads to severe inhibition of terminal branching. This inhibition is thought to be caused by downregulation of PTEN mediated by NEDD4-1. Indeed, decreasing PTEN in dysfunctional NEDD4-1 cells rescued branching defects [86]. Interestingly, it was also shown that NEDD4-1 ubiquitinated AMPA receptors, promoting their endocytosis [87]. A recent study associated polymorphisms in *NEDD4-1* gene with schizophrenia and cognitive dysfunction [88]. NEDD4-2 is called the E3 ligase of ion channels and transporters because it has been shown that, in Xenopus oocytes, it strongly inhibits the activity of several Nav channels. In cortical neurons, it controls the intracellular concentration of sodium by acting on voltage-gated channels. This was demonstrated in fetal cortical neurons from NEDD4-2 deficient mice [89]. A study in humans suggested a role for *NEDD4-2* gene in photosensitive generalized epilepsy, but this remains to be proven [90].

Overall, to date, three genes of the NEDD4 E3 ligases family (*ITCH*, *HECW2* and *NEDD4-2*) have been related to syndromic neurodevelopmental disorders. Interestingly, aside from neurodevelopmental features, macrocephaly appears to be a constant clinical manifestation. Of note, macrocephaly has also been observed in neurodevelopmental disorders related to other E3 ligase-encoding genes such as *HUWE1* [91]. As mentioned above, E3 ligases interact with PTEN and other proteins involved in the PI3K-AKT-mTOR signaling pathway. Pathogenic variants in several genes of this pathway lead to overgrowth syndromes with neurodevelopmental disorders and macrocephaly [92].

## 5. NEDD4 E3 Ligases in Neurodegeneration

More and more evidence indicates that defects in the ubiquitin–proteasome pathway initiate or contribute to the worsening of neurodegeneration in various neurodegenerative diseases. Exploring the roles of HECT E3 ligases (proteins highly expressed in neurons and participating in processes involved in neurodegeneration, such as protein aggregation, oxidative stress and apoptosis and abnormalities in glutamatergic transmission) has become important.

Protein aggregate formation is considered to be directly involved in the pathophysiology of many neurodegenerative diseases. Researchers have cited the aggregation of TDP-43 proteins in Amyotrophic Lateral Sclerosis (ALS), amyloid-beta in Alzheimer disease (AD), α-synuclein in Parkinson disease (PD), or polyglutamine-expanded Huntingtin protein in Huntington disease (HD) [93,94,95], for example. NEDD4-1 is implicated in targeting α-synuclein to the endosomal compartment and in lysosomal degradation of α-synuclein [96,97]. It has also been shown to protect against α-synuclein-induced toxicity in *Drosophila* and in rodent models of PD. Overexpression of NEDD4-1 in *Drosophila* brain rescue α-synuclein-induced locomotor defects [98]. Moreover, NEDD4-1 is implicated in Amyloid-β peptide regulation through P-glycoprotein ubiquitination [99]. A role for ITCH has also been indicated in several neurodegenerative diseases. It is found in polyglutamine-expanded huntingtin of ataxin-3 perinuclear aggregates and interacts with them. Its overexpression reduces aggregation of misfolded protein in cells under stress conditions [100]. ITCH, such as WWP1, another NEDD4 E3, interacts with spartin, a protein encoded by the *SPG20* gene which is mutated in an autosomal recessive form of hereditary spastic paraplegia [15]. ITCH, WWP1, as well as NEDL1, ubiquitinate and allow degradation of ErbB4 protein in a cell model of breast cancer [101]. Mutations in *ErbB4* gene, encoding a member of the epidermal growth factor receptor, disrupt the Neuregulin-ErbB4 pathway, causing Amyotrophic Lateral Sclerosis (ALS), a neurodegenerative disease characterized by the loss of upper and lower motor neurons [102]. Neurodegenerating motor neurons in ALS display TDP-43 positive aggregates containing the HECT E3 SMURF2 and some of its substrates, Smad2/3 [103]. NEDL1 was also associated with ALS caused by mutation of Superoxide Dismutase 1 *(SOD1)* gene. It was described as an E3 ubiquitin ligase, able to ubiquitinate and mediate proteasomal degradation of mutant, but not wild-type, SOD1 proteins [53]. Interestingly, mice overexpressing human *HECW1* gene encoding NEDL1 showed motor neuron degeneration and muscle atrophy, as observed in ALS [104].

Oxidative stress and activation of apoptotic pathways are involved in the pathophysiology of many neurodegenerative diseases. Oxidative stress produces reactive radical oxygen species (ROS), which trigger the expression of pro-apoptotic factors. Alzheimer’s disease (AD), PD and ALS has been associated with impaired insulin/insulin growth factor (IGF)-1 signaling [105]. IGF-1 degradation is mediated by the ubiquitin-proteasome system (UPS), and NEDD4-1 plays a key role in this process. NEDD4-1 is upregulated by a variety of neurotoxins that elicit oxidative stress in neurons, leading to IGF-1 degradation by the UPS. An elevated NEDD4-1 expression was found in brain tissues of AD, PD and HD patients, and also in the spinal cord tissues of ALS patients and mutant *SOD1* mice. Downregulation/inactivation of NEDD4-1 rescued neurons from death mediated by zinc toxicity [106]. NEDD4-1 has also been associated with other proteins that are particularly important in the regulation of cellular stress response (HSF-1) and apoptosis (NDFIP1). Heat Shock Transcription Factor-1 (HSF-1) is a master stress transcription factor which activates gene encoding for chaperones and anti-apoptotic proteins. Its dysregulation is thought to be implicated in neurodegeneration, especially in α-synucleinopathy. Under proteotoxic stress conditions induced by α-Synuclein, NEDD4-1 is the E3 ligase in neurons that ubiquitinates HSF-1 for further degradation by the proteasome. Aberrant degradation of HSF-1 involving NEDD4-1 could be an important molecular key mechanism underlying α-synucleinopathy and extensive neurodegeneration [107]. NEDD4-1 also interacts with NDFIP1 (NEDD4 family interacting protein), a trans-membrane protein with a protective role in a cell model of PD, helping reduce apoptosis and improving cell survival rate. This binding generates enhanced expression of NDFIP1 [108]. Loss of NEDD4-1 has been associated with elevation of RTP801, a pro-apoptotic protein sufficient and necessary to induce neuronal death in cellular and animal models of PD [109]. SMURF1 and SMURF2 are other HECT E3 ligases with links to apoptotic pathways. SMURF1 has been described as a Hirano Body (HB)-associated protein [110]. HB were first observed in patient suffering from ALS and PD, and then in AD. SMURF1 is upregulated by pro-inflammatory cytokines playing a role in apoptosis in CNS injury [111]. It has also been shown to inhibit p53-mediated apoptosis by stabilizing MDM2-MDMX complex that ubiquitinate p53 leading to its degradation [51]. SMURF2 has been described as a negative regulator of TGF-β signaling, a major actor in apoptosis regulation. Treatment with carbamate pesticide, carbofuran, leads to neurodegeneration by increased TGF-β signaling with a significant SMURF2 down regulation [112]. TGF-β signaling is increased, especially in AD, PD and ALS patients [113]. Another major actor in apoptosis is p53 protein. p53-mediated apoptosis has been directly involved in processes leading to neurodegeneration. Interestingly, the HECT E3 NEDL1 enhances p53-mediated apoptosis [114].

Glutamate is the most abundant excitatory neurotransmitter in the central nervous system. In AD, cognitive decline is due to synaptic impairment caused by the cleavage of the amyloid precursor protein into the pathogenic peptide amyloid-β (Aβ) [115]. Aβ decreases the subtype of ionotropic glutamate receptor AMPA-R at the membrane. The precise molecular mechanisms leading to this decrease remain unclear; however, in cultured neurons with Aβ-induced synaptic dysfunction, a role for ubiquitination mediated by NEDD4-1 on AMPA-R was identified. NEDD4-1 is known to target AMPA-R and Aβ promotes its recruitment, thus increasing ubiquitination and degradation of the synaptic receptors [116]. The HECT E3 NEDD4-2 is implicated in the ubiquitination and degradation of BEST1 (bestrophin-1), a calcium-activated chloride channel expressed at the surface of neurons and astrocytes [117]. BEST1 is implicated in glutamate and GABA release, associated with modulating neuronal excitability and synaptic transmission under pathological conditions such as neuroinflammation and neurodegeneration. Another link has been extensively described between glutamate and neurodegenerative diseases: glutamate excitotoxicity. Excessive glutamate in synapses is toxic and has been linked to AD, ALS and HD. Dysfunctional glutamate transporters contribute to this excitotoxicity [118]. The HECT E3 NEDD4-2 can mediate ubiquitination of glutamate transporters in vitro and in in vivo models of PD [119]. In MPP+ (1-methyl-4-phenylpyridinium) treated astrocytes, ubiquitinated (Ub) glutamate transporters GLT-1 levels are increased while non-Ub GLT-1 levels are reduced. This is reversed by siRNA-mediated knockdown of NEDD4-2. Similar results were obtained in the MPTP mouse model of PD (1-methyl-4-phenyl-1,2,2,6-tetrahydropyridine). Knockdown of NEDD4-2 in this mouse model resulted in improvement of movement disorders [120].

## 6. Conclusions and Future Perspectives

**Figure 3:**
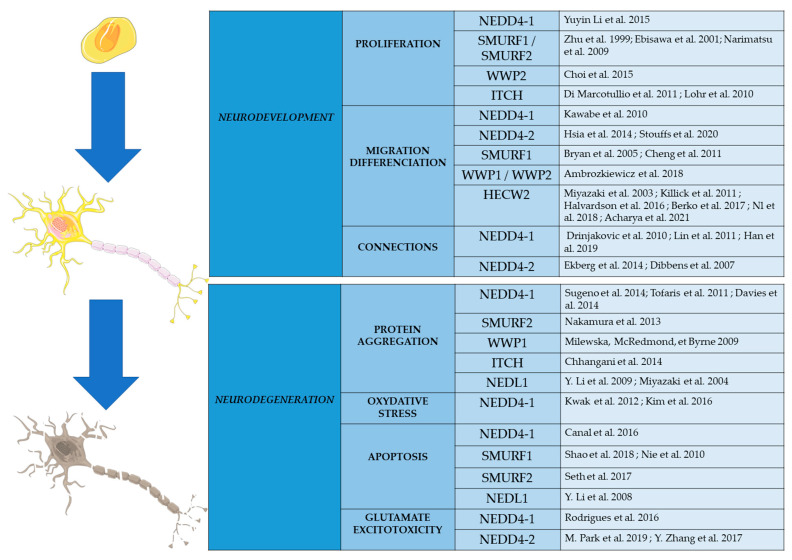
Schematic representation of the roles played by E3 ligases of the HECT subfamily in cellular processes involved in CNS development and neurodegeneration. Substrates of these enzymes are presented in the references.

The ubiquitin pathway is a major actor in the regulation of protein homeostasis and the activity of many proteins. The deregulation of this pathway, composed of many enzymes and particularly E3 ligases, leads to defects in neuronal development and function, causing neurodevelopmental or neurodegenerative diseases (Figure 3). In this work, we have provided the first review on the functions and regulation of a particular subfamily of E3 ligases highly expressed in the brain, the NEDD4 subfamily of E3 HECT ubiquitin ligases. It is the best-characterized subgroup of the 28 HECT-type enzymes [121]. The 9 members of this NEDD4 subfamily have been highly conserved during evolution in mammals, but also in non-mammals, such as *Caenorhabditis elegans* or *drosophila*. Proteins sharing the same structures and domains as NEDD4 proteins have been found in yeast *Saccharomyces cerevisiae* and *Schizosaccharomyces pombe* [17].

E3 enzymes of the NEDD4 subfamily are known to be highly expressed in the CNS. Recent studies indicate that they have diverse and important roles in the development and function of neurons. They also participate in the cellular processes involved in the regulation of cell survival and programmed cell death (Figure 3). Genetic studies have shown that some of the genes encoding these enzymes are mutated in particular neurodevelopmental and neurodegenerative diseases. It is very likely that further genetic studies, using next generation sequencing on large cohorts of patients, will show the involvement of this E3 family in other CNS pathologies.

Neurodegenerative diseases are known to be age-related diseases. Age can lead to modifications in concentration and activity of enzymes of the ubiquitin pathway. Changes in activity can be caused by post-translational modifications (PTM) such as deamidation. Indeed, deamidation is thought to be a molecular clock for protein turnover and can lead to protein denaturation or aggregation [122]. The effect of deamidation of ubiquitin ligases of the NEDD4 family should be, like phosphorylation, seriously studied. The modification of their concentration or activity could affect cellular processes and result in neurodegeneration.The regulatory mechanisms of NEDD4 are quite diversified, as seen previously. This opens interesting opportunities to develop therapeutics that would allow modulation (blocking, decreasing or increasing) of their actions. We could target the protein domains of regulation of the enzymatic activity, such as the HECT enzymatic domain, and the domain of interaction with ligands. Some molecules have been already developed to act on NEDD4 proteins, such as the anticancer drug Bortezomib, which interacts with several proteins of the NEDD4 subfamily [123]. Clomipramine, a drug used to treat depression, specifically blocks the HECT catalytic activity of the NEDD4 ITCH [124].

The NEDD4 subfamily has grown to be of great interest to those interested in physiological and pathophysiological processes in the CNS. Given the diversity and importance of the functions played by the proteins of this subfamily in neurons, and the possibility of developing therapeutics specifically targeting them, further research on these particular ligases is definitely needed.

## Data Availability

Not applicable.
